# Correction: Morroni et al. Antimicrobial Activity of Aztreonam in Combination with Old and New β-Lactamase Inhibitors against MBL and ESBL Co-Producing Gram-Negative Clinical Isolates: Possible Options for the Treatment of Complicated Infections. *Antibiotics* 2021, *10*, 1341

**DOI:** 10.3390/antibiotics11040464

**Published:** 2022-03-30

**Authors:** Gianluca Morroni, Raffaela Bressan, Simona Fioriti, Gloria D’Achille, Marina Mingoia, Oscar Cirioni, Stefano Di Bella, Aurora Piazza, Francesco Comandatore, Carola Mauri, Roberta Migliavacca, Francesco Luzzaro, Luigi Principe, Cristina Lagatolla

**Affiliations:** 1 Department of Biomedical Sciences and Public Health, Polytechnic University of Marche, 60126 Ancona, Italy; g.morroni@staff.univpm.it (G.M.); s.fioriti@staff.univpm.it (S.F.); gloria.dachille@gmail.com (G.D.); m.mingoia@staff.univpm.it (M.M.); o.cirioni@staff.univpm.it (O.C.); 2 Department of Life Sciences, University of Trieste, 34127 Trieste, Italy; rbressan@units.it (R.B.); clagatolla@units.it (C.L.); 3 Clinical Department of Medical, Surgical and Health Sciences, University of Trieste, 34129 Trieste, Italy; stefano932@gmail.com; 4 Unit of Microbiology and Clinical Microbiology, Department of Clinical-Surgical, Diagnostic and Pediatric Sciences, University of Pavia, 27100 Pavia, Italy; aurora.piazza@unipv.it (A.P.); roberta.migliavacca@unipv.it (R.M.); 5 Department of Romeo and Enrica Invernizzi Pediatric Research Center, Department of Biomedical and Clinical Sciences L. Sacco, University of Milan, 20157 Milan, Italy; francesco.comandatore@unimi.it; 6 Clinical Microbiology and Virology Unit, A. Manzoni Hospital, 23900 Lecco, Italy; c.mauri@asst-lecco.it (C.M.); f.luzzaro@asst-lecco.it (F.L.); 7 Clinical Pathology and Microbiology Unit, S. Giovanni di Dio Hospital, 88900 Crotone, Italy

## 1. Text Correction

Results have been implemented with additional data, which have been commented in the following paragraphs.

In Section 2.2.1, The sentence: “In contrast, MIC values for the six *Enterobacterales* ranged from 1 to 8 µg/mL, so zidebactam 0.5 µg/mL, which was a sub-inhibitory dose for all strains tested, was used for testing in combination with aztreonam.” [1] was changed to *Results—Checkerboard assays*—Section 2.2.1:

“In contrast, MIC values for the six *Enterobacterales* ranged from 1 to 8 µg/mL, so for testing in combination with aztreonam, zidebactam was used both at 0.5 µg/mL, which was a sub-inhibitory dose for all strains tested, and at a 1:1 ratio.”

In Section 2.2.1, At the end of the last paragraph, the following sentence was added: “Checkerboard assays testing the combination aztreonam/zidebactam at a 1:1 ratio confirmed previous results, showing synergistic effect against *C. amalonaticus* N18, *K. pneumoniae* KL 12 SG and *S. maltophilia* but not against the three *E. coli*, *K. pneumoniae* LC954/14, *C. indologenes* and *E.* meningoseptica (Table 1).” 

In Section 2.2.4, A correction has been made to *Results*—*Time-kill assays with aztreonam/zidebactam 1:1 ratio*—Section 2.2.4:

“Time-kill assays were also performed with aztreonam/zidebactam at 1:1 ratio against *C. amalonaticus* and *S. maltophilia*, while at 1:1 ratio was already synergistic in *K. pneumoniae* KL 12 SG with 0.5 mg/L concentration (Figure 3). Results were summarized in Figure S1. In *C. amalonaticus* synergies were also confirmed with aztreonam/zidebactam at 1:1 ratio of 1 mg/L. In *S. maltophilia*, although we did not detect synergies with 0.5 mg/L of zidebactam (regardless of aztreonam concentration), the two antibiotics showed synergistic effect with a 4 mg/L concentration, although the regrowth observed at 24 h suggests that this combination needs to be further analyzed in a higher number of *S. maltophilia* isolates.”

In Section 4.5, The sentence: “ZID, for which a recommended fixed concentration was not available, was tested in the range 0.125–32 µg/mL, alone and in association with ATM, by checkerboard assay in 96-well microtiter plates on an initial inoculum of 5 × 10^5^ CFU/mL. The combination was considered synergistic when the fractional inhibitory concentration index (FICI) was ≤0.5” was changed to *Materials and Methods—MIC evaluation and checkerboard assays*—Section 4.5:

“Zidebactam (ZID), for which a recommended fixed concentration was not available, was tested in the range 0.125–32 µg/mL, alone and in association with ATM, by checkerboard assay in 96-well microtiter plates on an initial inoculum of 5 × 10^5^ CFU/mL. Besides, as the Clinical Laboratory and Standards Institute (CLSI) in the United States of America recommends that testing of zidebactam in combination with cefepime is carried out at a 1:1 ratio, additional testing of the combination aztreonam/zidebactam at a 1:1 ratio was carried out. The combination was considered synergistic when the fractional inhibitory concentration index (FICI) was ≤0.5.”

In the section Supplementary Materials, Figure S1 was added. Figure S1 shows time-kill assays performed with aztreonam/zidebactam at 1:1 ratio against *C. amalonaticus* and *S. maltophilia*.

## 2. Error in Table 1

The MICs obtained using the combination aztreonam/zidebactam at a 1:1 ratio have been added in Table 1 (column #13).

The authors apologize for any inconvenience caused and state that the scientific conclusions are unaffected. The original publication has also been updated.

## Figures and Tables

**Table 1:** MIC (µg/mL) of aztreonam, alone and in association with BLIs, of tested strains.

Strain	Metallo-β-Lactamase	Serine-β- Lactamase	MIC ATM	MIC ATM after Addition of							MIC ZID	MIC ATM/ZID 1:1 Ratio	REF
				CLA ^a^	TAZ ^b^	SUL ^b^	VAB ^c^	AVI ^b^	REL ^b^	ZID ^d^			
***E. coli* CP-Ec3**	*bla*_VIM-1_	*bla*_KPC-2_	>32	>32	>32	>32	>32	8	>32	32	1	1	[16]
***E. coli* CP-Ec4**	*bla*_VIM-1_	*bla*_TEM-1_ * *bla*_CTX-M-15_ * *bla*_SHV-12_	>32	16	>32	>32	>32	32	>32	>32	1	1	[16]
***E. coli* 482483**	*bla*_NDM-5_	*bla*_TEM-1_ * *bla*_CTX-M-15_	>32	16	>32	>32	>32	8	>32	32	1	1	[17]
***C. amalonaticus* N18**	*bla*_VIM-1_	* *bla*_SHV-12_	>32	8	>32	>32	>32	0.25	4	0.5	4	0.5	[18]
***K. pneumoniae*** **KL 12 SG**	*bla*_NDM-1_	*bla*_TEM-1_ * *bla*_CTX-M-15_	>32	>32	>32	>32	4	0.25	4	0.25	8	0.5	This study
***K. pneumoniae* LC954/14**	*bla*_NDM-1_	* *bla*_CTX-M-15_ * *bla*_SHV-182_	>32	>32	>32	>32	8	0.25	4	>32	1	1	[19]
***C. indologenes* LC650/17**	*bla*_IND-3_	* *bla*_CIA_	>32	>32	>32	>32	>32	>32	>32	>32	>32	>32	[20]
***E. meningoseptica* LC596/11**	*bla*_B-9_ *bla*_GOB-13_	* *bla*_CME-1_	>32	>32	>32	>32	>32	>32	>32	>32	>32	>32	[20]
***S. maltophilia***	*bla*_L-1_	* *bla*_L-2_	>32	>32	4	8	2	1	0.5	0.5	>32	0.5	[20]

MIC, minimum inhibitory concentration; BLIs, β-lactamase inhibitors; ATM, aztreonam; CLA, clavulanate; TAZ, tazobactam; SUL, sulbactam; VAB, vaborbactam; AVI, avibactam; REL, relebactam; ZID, zidebactam. a: 2 µg/mL; b: 4 µg/mL; c: 8 µg/mL; d: 0.5 µg/mL. * ESBL.
