# Supplementary material for: Antimicrobial Activity of Aztreonam in Combination with Old and New β-Lactamase Inhibitors against MBL and ESBL Co-Producing Gram-Negative Clinical Isolates: Possible Options for the Treatment of Complicated Infections
Source: Antibiotics (Basel). 2021 Nov 3;10(11):1341. doi: 10.3390/antibiotics10111341 (PMC8615000; doi:10.3390/antibiotics10111341)
Supplement: Supplementary file 1 [file antibiotics-10-01341-s001.zip › antibiotics-1434588-Figure S1.pdf]

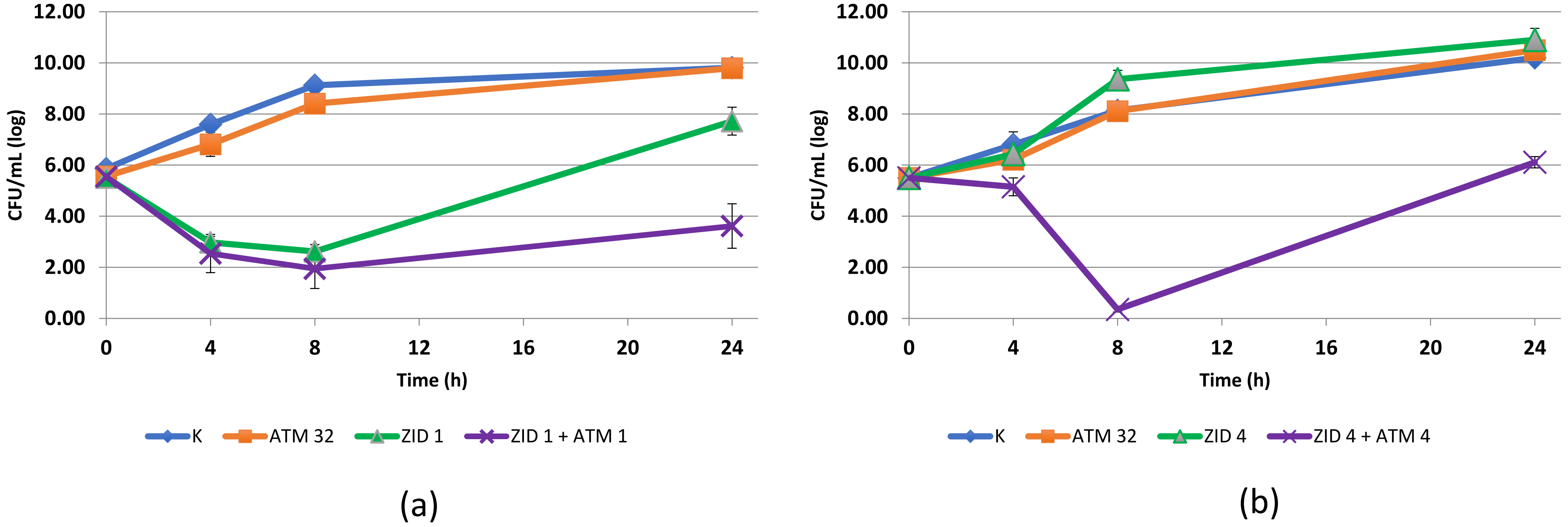

Figure S1. Twenty-four-hour time–kill curves of aztreonam (ATM) and zidebactam (ZID), alone and in 1:1 combination, on: (**a**) *C. amalonaticus* N18; (**b**) *S. maltophilia*. K: positive control (without the addition of antimicrobials). Mean values and standard deviation of three independent experiments are reported.
